# Photocontrol of Voltage-Gated Ion Channel Activity by Azobenzene Trimethylammonium Bromide in Neonatal Rat Cardiomyocytes

**DOI:** 10.1371/journal.pone.0152018

**Published:** 2016-03-25

**Authors:** Sheyda R. Frolova, Olga Gaiko, Valeriya A. Tsvelaya, Oleg Y. Pimenov, Konstantin I. Agladze

**Affiliations:** 1 Moscow Institute of Physics and Technology, Dolgoprudny, Russian Federation; 2 Institute of Theoretical and Experimental Biophysics, Russian Academy of Science, Pushchino, Russian Federation; Dalhousie University, CANADA

## Abstract

The ability of azobenzene trimethylammonium bromide (azoTAB) to sensitize cardiac tissue excitability to light was recently reported. The dark, thermally relaxed *trans*- isomer of azoTAB suppressed spontaneous activity and excitation propagation speed, whereas the *cis*- isomer had no detectable effect on the electrical properties of cardiomyocyte monolayers. As the membrane potential of cardiac cells is mainly controlled by activity of voltage-gated ion channels, this study examined whether the sensitization effect of azoTAB was exerted primarily via the modulation of voltage-gated ion channel activity. The effects of *trans*- and *cis*- isomers of azoTAB on voltage-dependent sodium (INav), calcium (ICav), and potassium (IKv) currents in isolated neonatal rat cardiomyocytes were investigated using the whole-cell patch-clamp technique. The experiments showed that azoTAB modulated ion currents, causing suppression of sodium (Na^+^) and calcium (Ca^2+^) currents and potentiation of net potassium (K^+^) currents. This finding confirms that azoTAB-effect on cardiac tissue excitability do indeed result from modulation of voltage-gated ion channels responsible for action potential.

## Introduction

A remote, easily reversible, and precise method for controlling excitable biological tissues, such as heart and neural networks, would have enormous potential for biomedical applications. The sensitization of ion channels to light provides a potential noninvasive tool to control the membrane potential and, therefore, the excitation of the cell. Cells may be sensitized to light either through genetic modifications (insertion of the light-sensitive proteins of microbial opsins, such as channelrhodopsin-2 or halorhodopsin [1]) or through application of light-responsive substances that alter the conductance of ion channels [2,3,4,5,6]. Such substances include azobenzene derivatives. [7]. Azobenzene is the most extensively used synthetic photo-switch, [8] and can easily be chemically modified. A dark, thermally relaxed *trans*- isomer of azobenzene adopts an extended configuration that is longer than a higher energy *cis-* or “bent” isomer. Illuminating azobenzene with near-ultraviolet (near-UV) light at wavelengths of ~365 nm leads to accumulation of the *cis*- isomer. Visible light irradiation at wavelengths preferred for absorption by the *cis*- isomer (λ > 440 nm) switches it back to the *trans*- isomer. Photoisomerization cycles can be repeated multiple times without detectable photodestruction or loss of responsiveness. By mixing light stimuli of two wavelengths, it is possible to obtain dynamically stable mixture of two isomers, maintaining constant concentration of the *trans-* isomer [9].

In general, azobenzene derivatives possess several features that make them attractive as prospective biological photoswitches:

- the absorbance spectra of the azobenzene isomers are different;
- photoisomerization is fast and can be easily completed in less than 1 min;
- the molecules are stable and allow many cycles of photoisomerization.

Several azobenzene derivatives have been custom synthesized for the photocontrol of the structure-function of peptides, nucleic acids, proteins and their ligands, as well as for the photocontrol of neuronal activity [8,10,11,12]. Research has shown that azobenzenes appeared to reversibly block the nicotinic acetylcholine and other receptors [3,13,14]. However, the precise mechanism of their interaction with ion channels and the cell membrane remains unclear. One detailed mechanism that has been proposed involves voltage-gated potassium (Kv) channels. The molecules were designed in such a way that the azobenzene core was linked to a tetraethylammonium (TEA) group on one side and a hydrophobic tail on the other side of the molecule [10,15]. Maleimide was selected as the active group for the channel complex binding [16] because it binds covalently to cysteine at position 422 of the neuronal K^+^ channel. Accordingly, the molecule maleimide-azobenzene-quaternary ammonium (Mal-Azo-QA) binds at such a distance from the channel pore that it could plug the opening of the channel in the elongated *trans-* form, while in the *trans-cis* transition, the molecule shortens and unplugs the channel [16]. In another study, acrylamide-azobenzene-quaternary ammonium (AAQ) compound had been designed aiming to covalently attach to the external TEA-binding site of wild-type Kv channels [16]. Instead, mechanistic studies revealed that AAQ diffused through a cell membrane due to its hydrophobic tail and bound noncovalently to the internal vestibule of the channel [3]. The substitution of AAQ for the more hydrophobic benzoyl-azobenzene-quaternary ammonium (BzAQ) resulted in better permeability through the cell membrane [17]. Conversely, increasing the polarity of the azobenzene derivative by replacing acrylamide with quaternary ammonium-azobenzene-quaternary (QAQ) ammonium prevented the passage of the ligand through the cell membrane [17]. The resulting QAQ ligand photosensitized not only Kv but also voltage-gated sodium (Nav) and calcium (Cav) channels that are structurally similar to Kv and sensitive to TEA [17,18].

Azobenzene photoswitches have also been reported to control heartbeat in the leech [19]. We have recently shown the possibility of photocontrol of excitation waves in cultured monolayers of cardiomyocytes by application of azobenzene trimethylammonium bromide (azoTAB) [9,20,21]. AzoTAB mediated sensitization allowed controlling propagation of excitation through the entire cardiomyocyte network either uniformly or in a preferred spatial pattern. The *trans-* isoform of azoTAB elicited the maximal effect, which affected 1) spontaneous electrical activity in the cardiac tissue; 2) propagation speed of excitation with a modest effect on the maximal captured frequency in the tissue, and 3) shape of action potential (AP) upstroke [20]. “Delayed” upstroke of the AP can be accounted for by suppression of voltage-gated sodium, potassium and/or calcium channels. The aim of the present study was, therefore, to find out whether ion channels underlying action potential generation might be involved in the azoTAB-mediated effect. For this purpose, we performed perforated whole-cell patch-clamp recordings from single ventricular cardiomyocytes derived from neonatal rats under various conditions (with *trans-* and *cis-* isoforms of azoTAB at visible and near-UV light, respectively). Our results show that *trans*- azoTAB suppresses voltage-depended sodium (INav) and calcium (ICav) currents, and potentiates net potassium current (IKv).

## Materials and Methods

AzoTAB was synthesized by ChemRar (Russian Federation). Amphotericine B and TEA were obtained from Wako Pure Chemical Industries, Ltd. (Japan). Collagenase type II, penicillin/streptomycin, L-glutamine, and cell culture media Hank`s Balanced Salt Solution (HBSS), Dulbecco’s Modified Eagle’s Medium (DMEM), Leibovitz’s L15 medium, Phosphate Buffered Saline (PBS) and Fetal Bovine Serum (FBS) were purchased from Gibco I Thermo Fisher Scientific Inc. (USA). Tetrodotoxin (TTX) has been kindly provided by Professor Y.M. Kokoz (Institute of Theoretical and Experimental Biophysics, Russian Academy of Science, Russian Federation). Unless otherwise stated, other chemical reagents were obtained from Helicon (Russian Federation). All solvents used were of analytical purity.

All the experiments conformed to the Guide for the Care and Use of Laboratory Animals, published by the United States National Institutes of Health (Publication No/85-23, revised 1996) and approved by the Moscow Institute of Physics and Technology, Life Science Center Provisional Animal Care and Research Procedures Committee (Protocol #A2-2012-09-02). Cardioectomy was performed on 1–2-day-old neonatal rat pups under isoflurane anesthesia, followed by enzymatic dissociation of cardiac myocytes as previously described [20]. The isolated cardiac cells were plated at low density (~2.8*104 cells/cm2) onto fibronectin-coated microscope cover slips for patch-clamp experiments. The cells were incubated in DMEM, supplemented with 10% FBS, 2 mM of L-glutamine, and 100 U/ml of penicillin/streptomycin at 37°C in 5% CO_2_. Twenty-four hours after isolation and plating, the cells were washed with warm PBS and cultured in DMEM with 5% FBS.

### Electrophysiology

Currents were recorded in single cardiomyocytes, which were unconnected to neighboring cells, using the perforated patch configuration. As a perforating agent, amphotericine B was used. A 0.5 M stock solution of amphotericine B was prepared in dimethyl sulfoxide (DMSO) and diluted in a corresponding pipette solution to a final concentration of 0.24 mg/ml [22]. Experiments were carried out at room temperature (22–24°C) on days 1 through 3 postplating.

A cover slip with cultured cardiac cells was placed in a recording chamber mounted on the stage of an Olympus IX71 inverted microscope table (Olympus Corporation, Japan). The chamber was continuously perfused with an appropriate bathing solution. The bathing solution used for recording INav and ICav currents contained 10 mM HEPES/NaOH, 90 mM NaCl, 20 mM TEA-Cl, 10 mM CsCl, 1.2 mM KH_2_PO_4_, 5 mM MgSO_4_, 2 mM CaCl_2_, 20 mM D-glucose, pH = 7.25 (270 mOsm). The pipette solution contained 10 mM HEPES/NaOH, 130 mM CsCl, 5 mM MgSO_4_, 5 mM EGTA, pH = 7.25 (285 mOsm) [23]. For the whole-cell recording of IKv currents, the bathing solution contained 10 mM HEPES/KOH, 108 mM NaCl, 5 mM KCl, 1.2 mM KH_2_PO_4_, 5 mM MgSO_4_, 2 mM CaCl_2_, 20 mM D-glucose, pH = 7.25, and the patch pipette was filled with a solution containing 10 mM HEPES/KOH, 130 mM KCl, 5 mM MgSO_4_, 5 mM EGTA, pH = 7.25 (285 mOsm). A 10 mM stock solution of azoTAB was prepared in corresponding bathing buffer and stored at room temperature with protection from light. For electrophysiological measurements, azoTAB at a final concentration of 100 μM was used, as described in our earlier study [20]. AzoTAB was applied by changing the bath solution in the recording chamber. The cardiomyocytes were pre-equilibrated in the azoTAB-containing solution for at least 3 min before electrical stimulation sequences were begun. Photoisomerization of *trans-* azoTAB to the *cis*- isomer was induced by illuminating with near-UV (~365 nm) light for 90 s according to our established methodology [20]. A UV-LED (Model: LC-L1V3, Hamamatsu, Japan) was used as a light source. The light was applied to the experimental chamber using an LED head unit (Series: L11921, Hamamatsu, Japan). The power density of the UV light was ~170 mW/cm2 and was measured with a laser power meter Nova II P/N 7Z01550 (Ophir Spiricon Europe GmgH, Darmstadt, Germany) at the end of the LED head unit.

Patch pipettes were pulled from borosilicate glass (BF150-86-10, Sutter Instrument, USA) with tip resistances of ~3 Megaohm (MΩ) when placed into the experimental solution. The pipette offset was corrected to zero just prior to the formation of a Gigaohm (GΩ) seal. After formation of the GΩ seal, the pipette capacitance was cancelled using the amplifier fast capacitance cancellation settings. Electrical access to the cell by perforation was indicated by the appearance of slow capacitance currents that increased the amplitude and rate of decay when more amphotericin B pores formed in the membrane enclosed by the patch pipette. The access resistance was monitored using the slow whole-cell capacitance cancellation settings on the amplifier. Once the access resistance decreased below 12 MΩ, the experiment was started. Series resistance was compensated, if required.

Voltage clamp stimulation protocols were generated by pCLAMP 10.2 software (Molecular Devices, USA). Membrane currents were recorded using an Axopatch 200B patch-clamp amplifier, digitized by Digidata 1440A (both from Axon Instruments, Inc., USA), and the data were stored on a personal computer. Na^+^ channel currents were recorded from holding potentials (HP) of -80 mV during linear voltage ramps from -120 mV to +50 mV over a 200-ms period. A prestep from -80 mV to -120 mV for 100 ms was applied [19,24]. The voltage-dependence of the peak Na^+^ currents was determined by measuring peak inward currents for cells depolarized from -80 to +50 mV in 10-mV increments. The effect of azoTAB on L-type Ca^2+^ currents (ICa-L) was analyzed using a CsCl-rich solution and TEA chloride to suppress K^+^ currents. To study ICa-L without contamination from Na^+^ currents, a 100-ms prepulse to -40 mV from a HP of -80 mV was applied [25,26]. The voltage dependence of the peak ICa-L was assessed using depolarizing pulses from -80 mV to +40 mV in 10 mV increments, which were applied for 300 ms. The peak ICa-L was measured at 0 mV. Outward IKv was elicited by a 300-ms depolarizing pulse from 0 mV to +60 mV (HP of -70 mV). The amplitude of the IK was measured at the end of the voltage step. Current/voltage (I/V) relationships were plotted using the peak values of the sodium (INav, peak) and calcium (ICavpeak) currents and the steady-state values of the K^+^ currents (IKss). In order to compensate for variations in cell membrane surface size, the currents were divided by the cell membrane capacitance and expressed as the current density (pA/pF). Typically, the membrane capacitances measured with pCLAMP 10.2 software ranged from 5–40 pF.

### Data analysis

The data analysis was performed using Clampfit 10.2 (Molecular Devices, USA) and SigmaPlot 12.0 (Systat Software, USA) software. Numerical values are given as the mean ± SEM and averaged from at least three neonatal cardiomyocytes from independent cell isolations. Statistical significance was evaluated by the Student’s *t*-test, with a value of *p<* 0.05. The safety factor (SF) for conduction (S1 File. Supporting information) was calculated with the program Mathematica (Wolfram, USA) using following equation [27]

$$\text{SF}=\frac{\int_{APD}I_{Na}*\text{dt}+\int_{APD}I_{Ca}*\text{dt}}{\int_{APD}I_{K}*\text{dt}+{\text{Q}}_{c}}$$

where INa and ICa are the axial currents out of the cell, IK is the axial current in the cell, QC is the integral of transmembrane current over time. Thus, an SF > 1 indicates the margin of safety, whereas the SF < 1 indicates that the charge requirements are not met and conduction fails [27].

The concentration-response curve was fit to the equation

$$\text{Y}=100*[{\left(X\right)}^{n}/({\left(X\right)}^{n}+{\left(\text{E}{\text{C}}_{50}\right)}^{n})],$$

where X is the azoTAB concentration, EC_50_ is the azoTAB concentration required for a 50%inhibition, and n is the slope of the curve.

## Results

**Fig 1:**
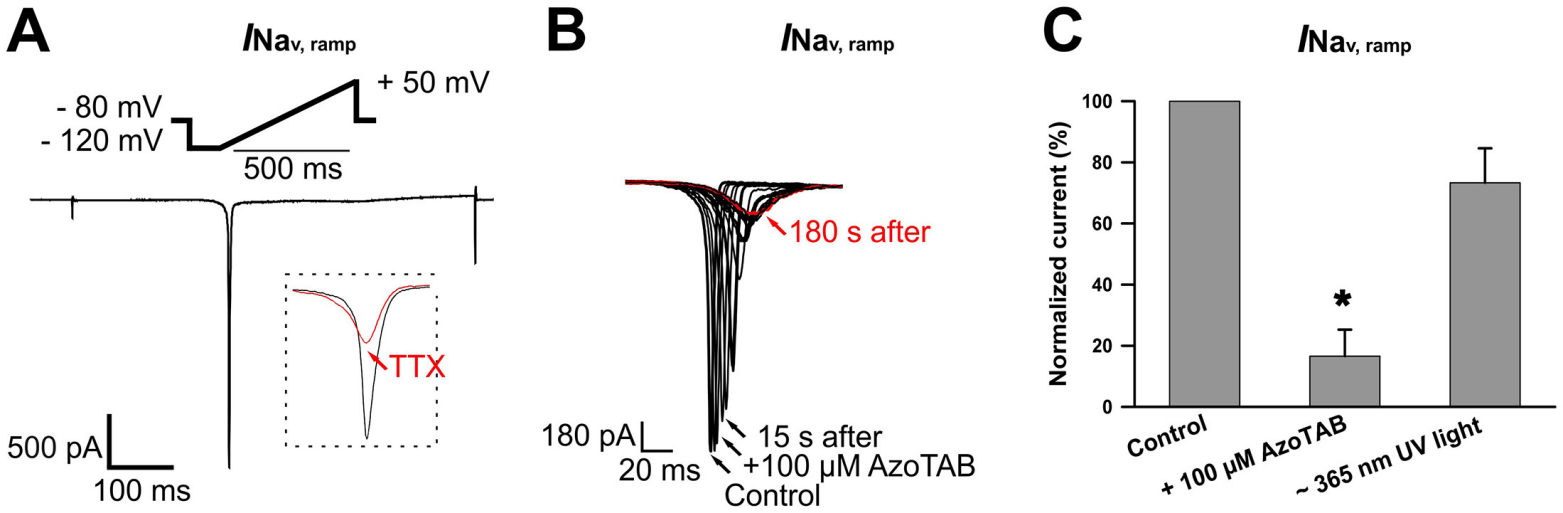
Effect of azobenzene trimethylammonium bromide (azoTAB) on ramp currents in neonatal rat ventricular myocytes. (A) A representative TTX-sensitive current that was evoked when the voltage was increased smoothly from -120 to +50 mV for 200 ms. The cell was prepulsed to -120 mV for 100 ms from a HP of -80mV. The voltage protocol is shown above the current trace. The inset shows scaled current traces for comparison before and after the addition of 10 μM TTX. Similar results were obtained in three other cells. (B) Scaled ramp-evoked currents recorded in response to the same ramp protocol (from -120 to +50 mV, 200 ms) in the control and after the addition of 100 μM *trans*- azoTAB. Currents were recorded every 15 s after the application of the photoreactive substance. Three minutes after the application, the current was inhibited by approximately 83% relative to that of the control. Similar results were obtained in three other cells. (C) Ion currents recorded before and after incubation with 100 μM *trans*-azoTAB, as well as after near-ultraviolet (near-UV) irradiation and expressed as percentage. Each cardiomyocyte was incubated in the presence of azoTAB at room temperature for at least 3 min in a measuring chamber. Near-UV was applied for 90 s. The data represent the means ± SEM from four cardiomyocytes, *p< 0.05.

Fig 1A shows a representative ramp current recorded under control condition. The shape of the voltage ramp is shown above the current trace. To inhibit this current, 10 μM TTX was required (inset in Fig 1A), confirming that it was a Na^+^ current. The behavior of the TTX-sensitive sodium current evoked by voltage ramps in the presence of 100 μM *trans*- azoTAB is demonstrated in Fig 1B. As shown in the figure, the ramp current clearly started to decrease after the application of azoTAB. Approximately 3 min after the application, the current was inhibited by ~83% (*n* = 4) relative to a control response. The ramp current significantly recovered following exposure to near-UV irradiation, which converted *trans*- azoTAB to its *cis-* isomer. The inhibition of ramp currents by the *trans-* isomer of azoTAB was also relieved after a long washout (data not shown). Normalized ramp currents are shown in Fig 1C.

**Fig 2:**
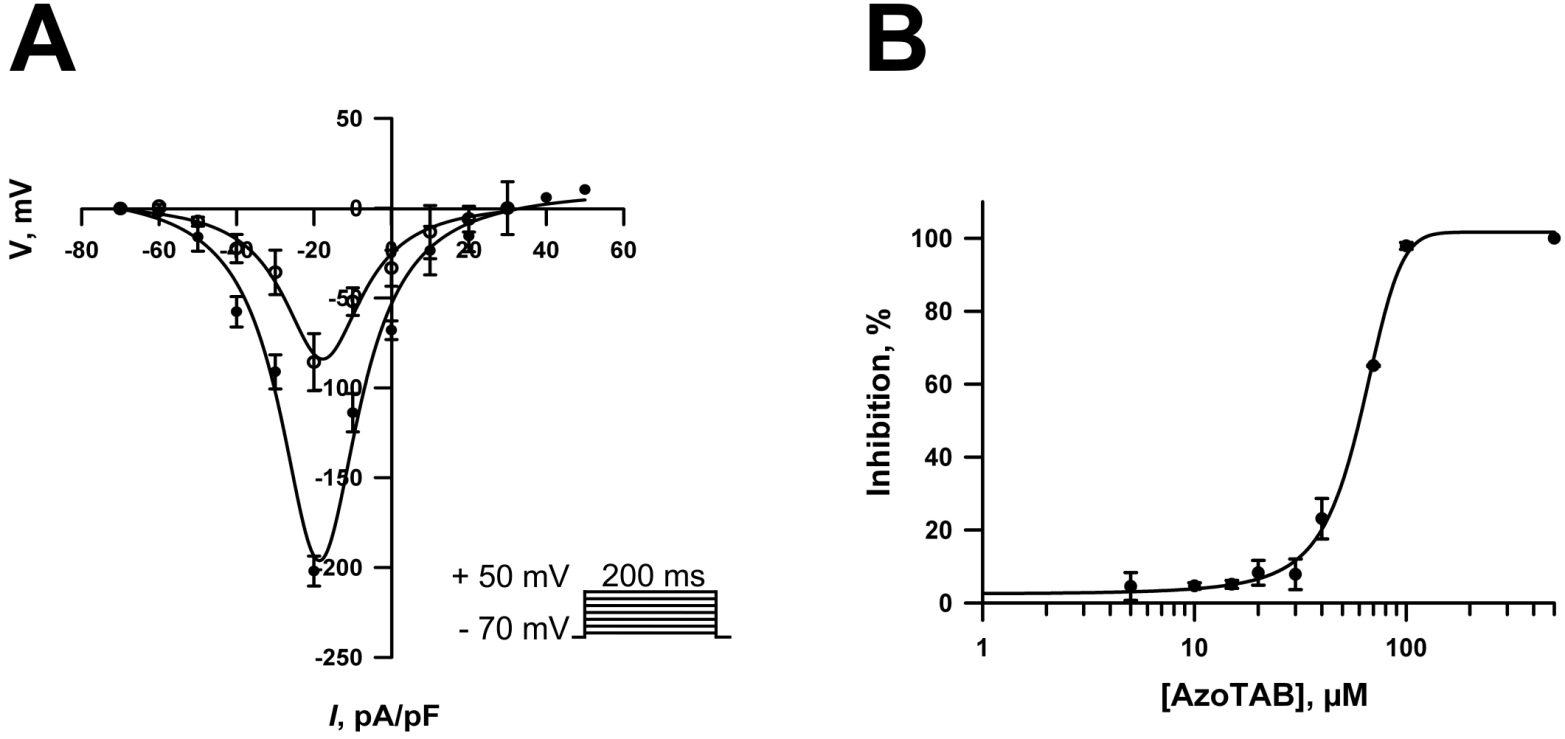
Effect of azoTAB on voltage-dependent Na^+^ currents in neonatal rat ventricular myocytes. (A) Current-voltage relationships recorded from single neonatal ventricular cardiomyocytes under control conditions (filled circles) and after exposure to 100 μM *trans*-azoTAB (open circles). Inset: the shape of the current-voltage stimulation protocol. The current density was calculated as the Na^+^ peak current divided by the membrane capacitance of each cell (*n* = 4). (B) Concentration dependency for *trans*- azoTAB-induced inhibition of INav in neonatal rat ventricular cardiomyocytes. Mean ± SEM, *n* = 3–4 for each point.

We compared INav, peak elicited in response to 200-ms depolarizing pulses at various potentials ranging from -80 mV to +50 mV (HP = -80 mV) in control and in presence of 100 μM *trans*- azoTAB at visible light (Fig 2A, inset). The INa peak currents expressed as current density were plotted versus membrane voltage to obtain an activation current-voltage (I/V) curve (Fig 2A). Averaged I/V relationships in Fig 2A show that under control conditions, INav developed at potentials positive to -50 mV and attained the maximum value at -20 mV. When azoTAB was applied, the current density of INav was reduced by approximately 60% without significant changes in the shape of the I/V curve (Fig 2A). The relationship between the azoTAB concentration and the percentage inhibition of INav was determined and is shown in Fig 2B. As demonstrated in Fig 2B, *trans*- azoTAB suppressed INav amplitude in a concentration-dependent manner with EC50 = 60 ± 3.9 μM (n = 3–4 for each point).

**Fig 3:**
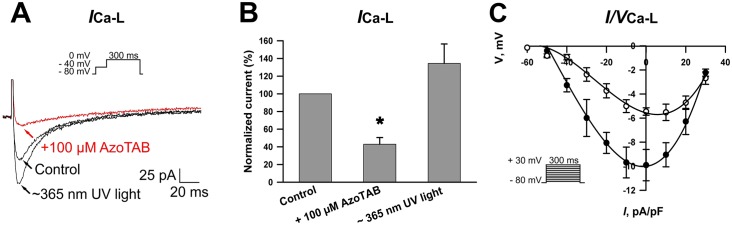
Effect of azoTAB on voltage-dependent Ca^2+^ currents in neonatal rat ventricular cardiomyocytes. (A) L-type Ca^2+^ currents obtained in the absence (control) and presence of 100 μM *trans*- azoTAB and after ~365 nm near-UV irradiation. Inset: original current trace in response to a voltage step from -40 to 0 mV for 300 ms. Inactivation of INav was achieved by a prestep from a holding potential HP of -80 mV to -40 mV for 100 ms. Similar results were obtained in three other cells. (B) ICavpeak recorded before and after incubation with 100 μM azoTAB, as well as after near-UV irradiation, and expressed as a percentage of that of the peak currents before the treatment. Each cardiomyocyte was incubated in the presence of azoTAB at room temperature for ~3 min in a measuring chamber. The currents were inhibited by approximately 60% relative to the control. Near-UV was applied for 90 s. The data are the means ± SEM from three cardiomyocytes, *p< 0.05. (C) Averaged I/V relations of the L-type Ca^2+^ currents elicited by the voltage-clamp protocol illustrated in the inset (HP = -80 mV) and plotted before (filled circles) and after (open circles) the application of azoTAB. The values are expressed as the mean ± SEM, *n* = 4. The current density is plotted as a function of the voltage.

Fig 3A shows representative ICa-L traces under different conditions. The voltage-step protocol for eliciting ICa-L is shown as an inset. Similar to the voltage-dependent sodium currents studies, we compared ICavpeak generated by cardiomyocytes after treatment with 100 μM azoTAB at visible and near-UV light relative to the control (Fig 3B). The *trans*- azoTAB caused a decrease in the ICavpeak of ~60% (*n* = 3) compared to the control (Fig 3B). After near-UV light irradiation, the ICa-L, peak recovered (Fig 3B). Fig 3C shows the current-voltage relationships for ICavpeak in control and in the presence of *trans*- azoTAB. In the presence of *trans*- azoTAB, the current density of the ICavpeak was not shifted relative to that of the control.

**Fig 4:**
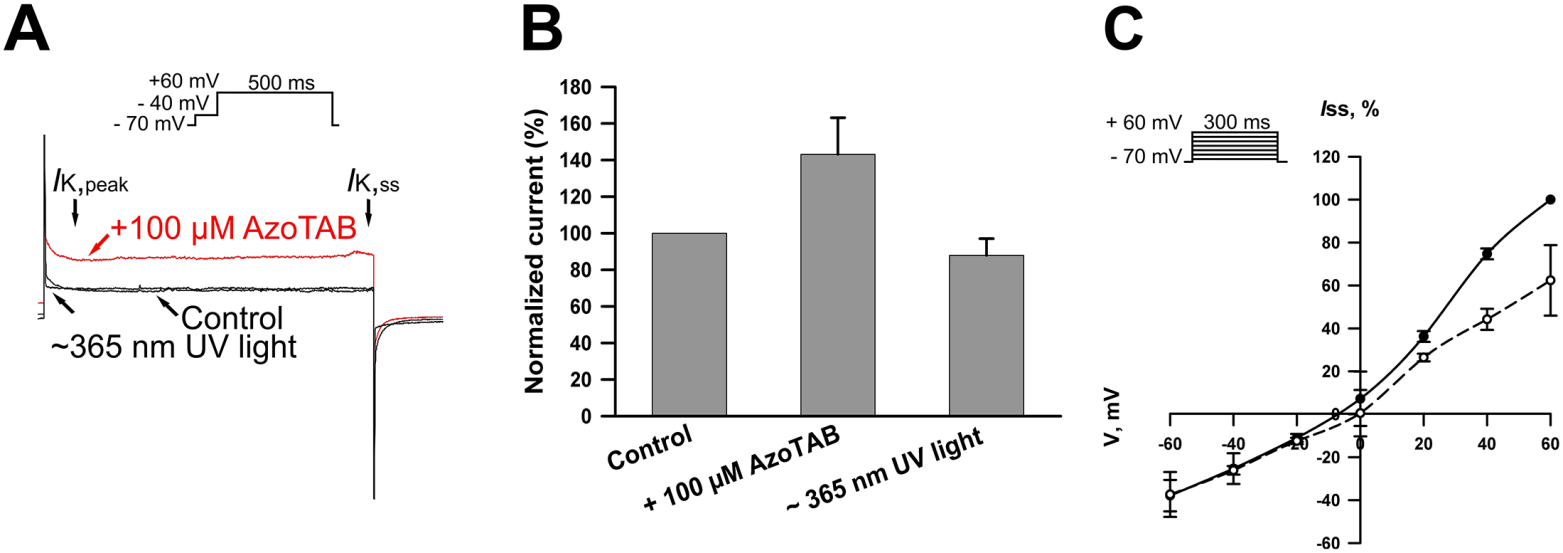
Effect of azoTAB on voltage-dependent K^+^ currents in neonatal rat ventricular cardiomyocytes. (A) Whole-cell outward K^+^ currents of the control in response to 300 ms depolarizing voltage steps from -70 mV to +60 mV, after application of 100 μM azoTAB under visible light (*trans*- azoTAB), and after ~365 nm UV irradiation. (B) Steady-state K^+^ currents recorded before and after treatment with 100 μM azoTAB, as well as after near-UV irradiation, and expressed as a percentage of the control IKss. Each cardiomyocyte was incubated in the presence of azoTAB at room temperature for ~3 min in a measuring chamber. Near-UV was applied for 90 s. The data are the means ± SEM from four cardiomyocytes, *p< 0.05. **C**. Averaged steady-state I/V curves in the control (filled circles) and after near-UV irradiation (triangle). The I/V curves were normalized to those of the control current, measured at +60 mV.

To determine whether azoTAB affected the outward K^+^ currents in neonatal rat ventricular cardiomyocytes, 100 μМ azoTAB were added to the external bath solution under visible light. The raw current traces in Fig 4A demonstrate that unlike INav and ICav, the extracellular application of *trans*- azoTAB potentiated IKv. However, near-UV irradiation reduced the steady-state potassium current back to the value of the control. The currents are presented in Fig 4B in percentages relative to those of the control. The current-voltage relationships for IKss of the control and after irradiation with near-UV are presented in Fig 4C.

## Discussion

The main purpose of this study was to clarify whether the previously observed inhibition of excitation waves in cultured monolayers of cardiomyocytes [9,20] was caused by modulation of voltage-dependent sodium, calcium and/or potassium currents by azoTAB. The results presented here show that azoTAB produces reversible suppression of INav, as well as ICav and potentiation of IKv. The azoTAB-effect is reversible, and INav, ICav and IKv can be restored to the control level after exposure to near-UV irradiation or a long washout (data not shown).

On the basis of the approach of others [27], we defined the safety factor to evaluate the influence of azoTAB on conduction at different conditions: in control (without azoTAB), in the presence of *trans*- and *cis*- azoTAB (after near-UV illumination). In our case, SF is equal to 1.80 under control conditions, SF = 0.15 in the presence of 0.1 mM of *trans*- azoTAB, and SF = 1.40 after near-UV illumination. The obtained results demonstrate that a decrease in membrane excitability causes a decrease of SF for electrical conduction after application of *trans*- azoTAB.

There are studies that show that Quaternary ammonium (QA) compounds block Na^+^ channels preferentially from the internal side [28,29]. External application of amphipathic QA compounds is far less effective, if only QA compounds do not contain a long hydrophobic tail to cross the membrane [30]. The quaternary lidocaine derivative, QX-314 (2-(triethylamino)-N-(2,6-dimethylphenyl)-acetamide) induces internal (cytoplasmic) pore blockade of single cardiac Na^+^ channels [31] and can also affect K^+^ and Ca^2+^ channels [31]. However, there are few compounds among azobenzene derivatives with quaternary ammonium that block INa^+^ and ICa^2+^. Among all tested photochromic ligands (PCLs) [17], only one with two QA groups seems to block Nav. When QAQ compound was applied into the cytoplasm through a patch pipette, it blocked most of the Na^+^ current in the *trans-* configuration. In contrast, extracellular application of QAQ failed to block Na^+^ currents [32]. QAQ resembles tertiary amine lidocaine and its derivative QX-314 [32]. In the case of azoTAB (0.1 mM concentration), however, INav and ICav decreased and IKv increased after application of the substance. The current densities of INav and ICav were reduced by approximately 60% in both cases without significant changes in the shape of I/V curves and reversal potentials, indicating that inhibition of INav and ICav is not associated with the change in membrane potential and ion permeability. Relatively fast washing out of cells from azoTAB terminated this suppression/activation. The possibility of wash out indicates that azoTAB does not bind covalently to the channels. The azoTAB molecule has a trimethylammonium group. Tetramethylammonium (TMA^+^) is well-known blocker of sodium channels [33]. Perhaps this is an explanation of decreasing of sodium currents.

Some anesthetics block channels, especially voltage-dependent sodium channels, by changing elasticity of the membrane [32]. AzoTAB, as a known surfactant, may also affect lipid bilayers [34]. A previous study demonstrated that azoTAB not only changed the stability of lipid vesicles but also destroyed these vesicles [34]. The *trans*- isomer of azoTAB is a linear molecule with a hydrophobic tail, which tends to penetrate the lipid membrane of the vesicle. The c*is*- isomer of azoTAB is bent, and the molecule is more polar and more water soluble than its *trans-* form. Thus, while in the *trans-* form, azoTAB can predominantly dissolve in lipid membranes; the *cis-* form of azoTAB dissolves in water solution. This finding is supported by the previous measurements of washout and binding of *trans-* and *cis*- isomers to the cell surface [20,21].

There are also studies in which photoresponsive surfactants were applied in order to control protein structure, including secondary [35], tertiary [36,37] and quaternary structure [38], along with the DNA condensation [39]. The ability to control the structure-dynamics-function relationship of proteins with light illumination using azoTAB was examined with bovine serum albumin (BSA) [36] and lysozyme [37]. In the presence of *trans*- azoTAB, BSA unfolded mainly in the α-helices region, while β structures were largely unaffected, and lysozyme exhibited a partially unfolded structure with a more exposed active site, which led to enhanced activity. The direct measurement of lysozyme dynamics indicates that this increased activity might be the result of enhanced internal motions induced by *trans*- azoTAB.

The measured in our study dose dependent response (Fig 2B) has a shape, which could not be explained by the competitive inhibition process. It has a sharp increase of the inhibition rate at the concentration interval 30–100 μM. It may indicate, that there is a complex cooperative inhibition of the ion channel activity, which either involves cooperative binding to several active sites or that the channel blockage is influenced by the structural changes in the cell membrane. We may conclude that further studies are needed to determine exact molecular mechanisms underlying azoTAB-effect. For instance, we hope that low angle neutron scattering of the crystallized ion channel proteins with and without azoTAB may give us the necessary information, however such a study goes far beyond the work presented.

## Conclusion

The present study shows that azoTAB in its *trans-* form suppresses fast Na^+^ and L-type Ca^2+^ currents and potentiates K^+^ currents. The transition of *trans-* azoTAB to *cis-* azoTAB obtained by UV irradiation leads to the recovery of normal channels functions.

## Supporting Information

S1 File Supporting information software. SF.nb (Wolfram Mathematica), INa and IK (control, azoTAB, UV).xlsx, ICa (control, azoTAB, UV).xlsx used for SF computation.
(ZIP)
